# The Most Influential Publications Regarding Hair Transplantation: A Bibliometric Review

**DOI:** 10.1007/s00266-024-04049-3

**Published:** 2024-09-13

**Authors:** Juan J. Lizardi, Dylan Treger, Savannah C. Braud, Tanya Boghosian, Rawan El Abd, Sinan K. Jabori, Seth R. Thaller

**Affiliations:** 1 University of Miami Leonard M. Miller School of Medicine, Miami, USA; 2 Florida Atlantic University Schmidt College of Medicine, Boca Raton, USA; 3 Washington University School of Medicine, St. Louis, USA; 4 Division of Plastic and Reconstructive Surgery, McGill University Health Centre, Montreal, Canada; 5 Division of Plastic Surgery, Dewitt Daughtry Department of Surgery, University of Miami Hospital, 1600 NW 10th Ave #1140, Miami, FL 33136 USA

**Keywords:** Hair transplantation surgery, Follicular unit extraction, Follicular unit transplant, Bibliometric review

## Abstract

**Introduction:**

This bibliometric review aims to assess the impact of significant publications within the field of hair transplantation. Citation counts will serve as a primary influence indicator.

**Methods:**

An exhaustive search was conducted using Clarivate’s Web of Science database, yielding 260 publications related to hair transplantation. These were evaluated and sorted based on citations, narrowing down to the 50 most highly cited works for analysis. Parameters including citation density, authorship, institutional affiliations, country of origin, year of publication, article topic, and the level of evidence for each publication were obtained.

**Results:**

Analyzed publications were cited a total of 1341 times. Authorship analysis revealed that the most significant contributors regarding hair transplantation were Bernstein and Rassman. We also identified the leading institutions affiliated with these works, highlighting the primary academic and research centers driving the field. Geographical analysis exhibited the US' dominance in producing impactful publications. Most publications were also classified within Level IV and Level V according to the Oxford Levels of Evidence system.

**Conclusion:**

This review provides a comprehensive snapshot of the pivotal publications shaping hair transplantation. Our findings underscore significant contributions within this field and may assist researchers and clinicians in understanding the evolution and the current state of the hair transplantation literature. This bibliometric analysis can serve as a roadmap for those seeking to delve into this rapidly evolving field, facilitating the identification of research gaps and formulating future research directions.

**Level of Evidence V:**

This journal requires that authors assign a level of evidence to each article. For a full description of these Evidence-Based Medicine ratings, please refer to the Table of Contents or the online Instructions to Authors www.springer.com/00266.

## Introduction

Hair transplantation surgery is a popular cosmetic surgery used to combat hair loss and enhance hair appearance. From its inception in the 1950s, the procedure has not only gained tremendous popularity but has also naturally evolved with various techniques. The original procedure, Punch Hair Transplantation, involved "punching out" sections of hair follicles—hence the name—for transplantation [1]. In the 1990s, a more sophisticated technique emerged known as the follicular unit hair transplant (FUT). This method involved excising hair-bearing strips from the donor, dissecting follicular units, and then transplanting these units to the desired site on the patient. Furthermore, follicular unit extraction (FUE), the most recent of the techniques, involves the precise and meticulous extraction of individual hair follicles, which are then implanted to complete the transplantation [2–5].

Since its introduction and subsequent rise in popularity, FUE has become a sought-after option for patients desiring hair transplantation. That said, both FUT and FUE have proven effective, though each possesses its own set of advantages and disadvantages. For instance, FUE gained popularity partly because it offers similar outcomes without leaving significant scars. However, being a more detailed procedure, it is also more time-consuming, might necessitate multiple sessions, and can be more expensive. FUT alleviates some of these disadvantages, but patients might face a longer post-operative recovery period and have a more noticeable scar. As with all surgical procedures, both come with inherent risks and benefits tailored to individual patient characteristics. Aspects such as patient age, area of concern, hair styles, and scalp laxity should also be factored in [6].

The evolution of hair transplantation in the form of advanced techniques, instrumentation, and improved patient outcomes has triggered a surge in publications on the subject. This influx in research has equipped the scientific community with profound insights into the past and potential future of hair transplantation. To discern the trajectory of upcoming advancements in the field, one might consider executing a bibliometric analysis of the existing literature. A bibliometric review involves categorizing, quantifying, and appraising the research related to a specific topic to extract insights about it [7]. In the realm of hair transplantation surgery, a bibliometric analysis can spotlight the most influential and frequently cited publications.

The aim of this study is to conduct a bibliometric review of the most referenced publications on hair transplant surgery. The number of citations a publication garners will be indicative of its influence in the realm of hair transplantation. Secondary characteristics of these top-cited publications will be catalogued and examined to furnish a thorough overview of the discipline. By doing so, future clinicians, surgeons, and researchers can gain insights into the pivotal contributions to the domain and anticipate potential future developments.

## Methods

On April 7, 2023, Clarivate’s Web of Science database was used to obtain the data that were analyzed in this study. Search query “follicular unit transplantation” OR “follicular unit extraction” AND “hair” under all topics was used and yielded a total of 260 publications. Only publications available in English were analyzed. Publications were then sorted via number of citations, in descending order from highest to lowest count. Then, the data were exported to an Excel sheet for further analysis. On the offline Excel sheet, each publication was reviewed and included in the final list only if it fell under the scope of the project. In order to be included in the top 50 analysis, the publications had to pertain to hair transplantation surgery. Once the 50 most-cited publications were ascertained, they were moved to a separate sheet for further review.

Initially, the PubMedID’s of the 50 publications included in the review were copied and searched under the Web of Science database. This search query was analyzed by the citation report function to provide an overview of the 50 publications. Affiliated institutions, country of origin, authorship, year of publication, and publishing journal were also obtained for each of the publications. Lastly, each publication was reviewed and assigned a Level of Evidence (LOE) and a classifying topic. LOE was determined by Oxford’s level of evidence rating and was determined as follows:

1 = Systematic reviews of randomized trials or systematic reviews of inception cohort studies.

2 = Systematic reviews of cohort studies, inception cohort studies, cross-sectional studies, randomized trials, or observational studies with dramatic effect.

3 = Cohort studies (primarily retrospective), epidemiological/observational study.

4 = Case-control studies, low-impact cohort studies, animal trials.

5 = Simulations, models, or mechanism-based reasoning.

Classification of article topics was determined by the authors of the papers as follows: alternative use, anatomy/biology/basic science, expert opinion, novel approach, outcome, population statistics, review, surgical technique, and/or treatment guidelines. Lastly, publications were also assessed for their citation density, which was the frequency cited per year since being published.

VOSviewer was used to create visuals for co-occurrence map of author keywords and co-authorship maps of authors. Analysis was conducted using a Text (.txt) file that was exported from the Web of Science database. Text file was imported into VOSviewer (version 1.6.19), a software for constructing and visualizing bibliometric networks. Overlay visualization was used to show trends over time, with different colors indicating the different years in which the terms occurred. Larger the size of a term’s label and circle, the greater its weight or frequency. Similarly, thicker lines indicate stronger links, or degrees of correlation, between terms.

## Results

**Fig. 1:**
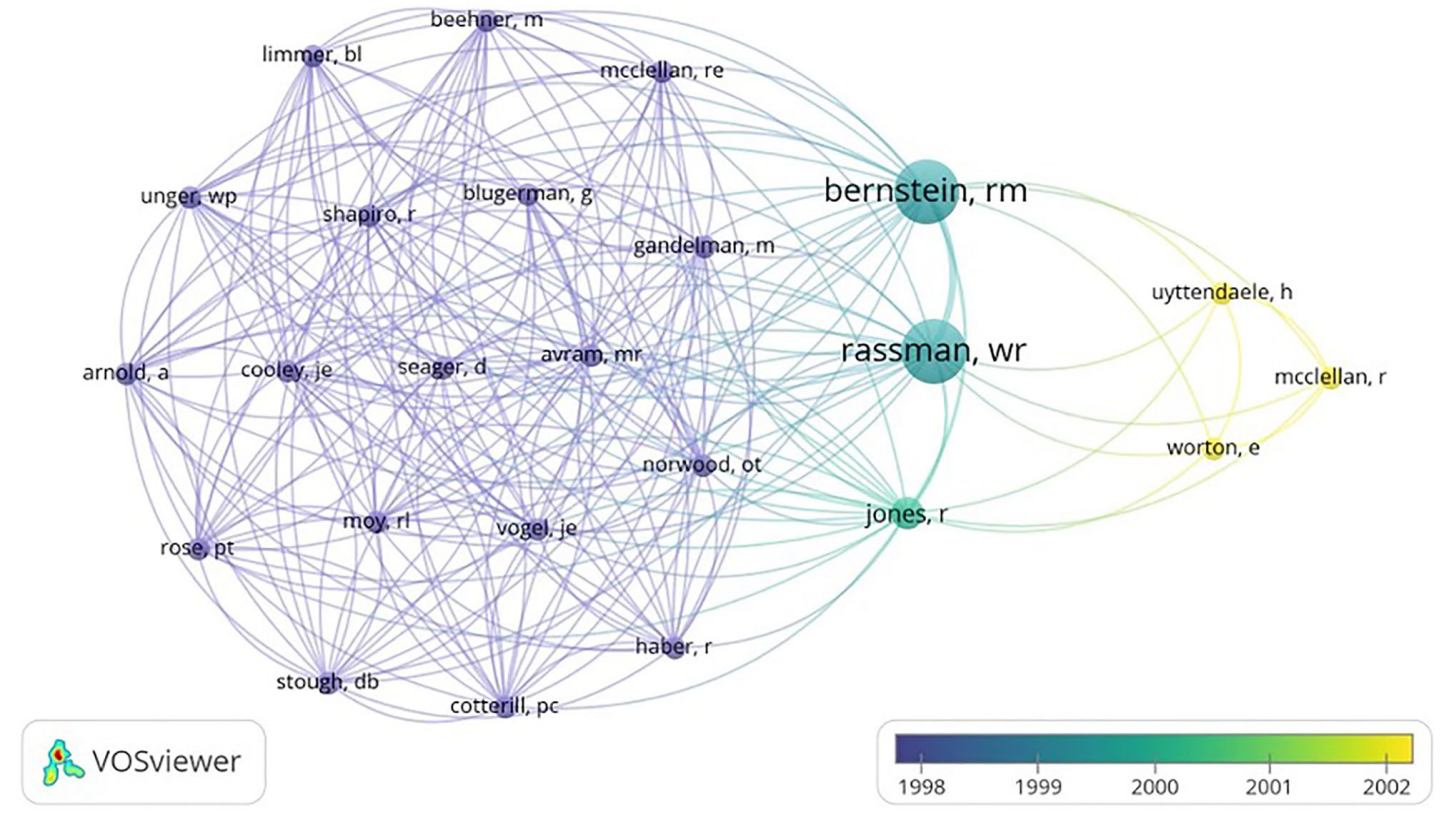
Co-authorship map of authors related to hair transplantation with overlay visualization

**Fig. 2:**
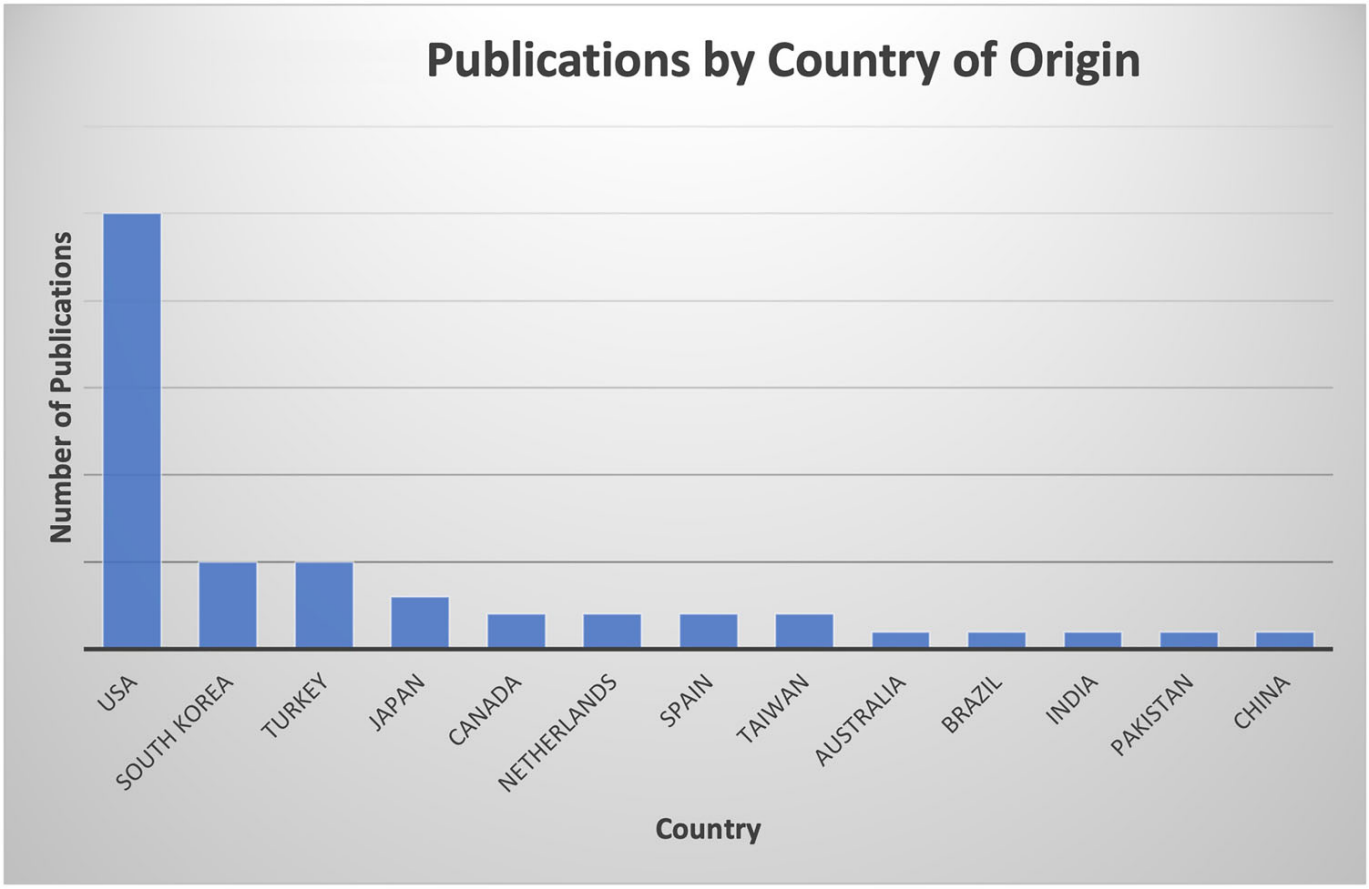
Publications by county of origin

**Fig. 3:**
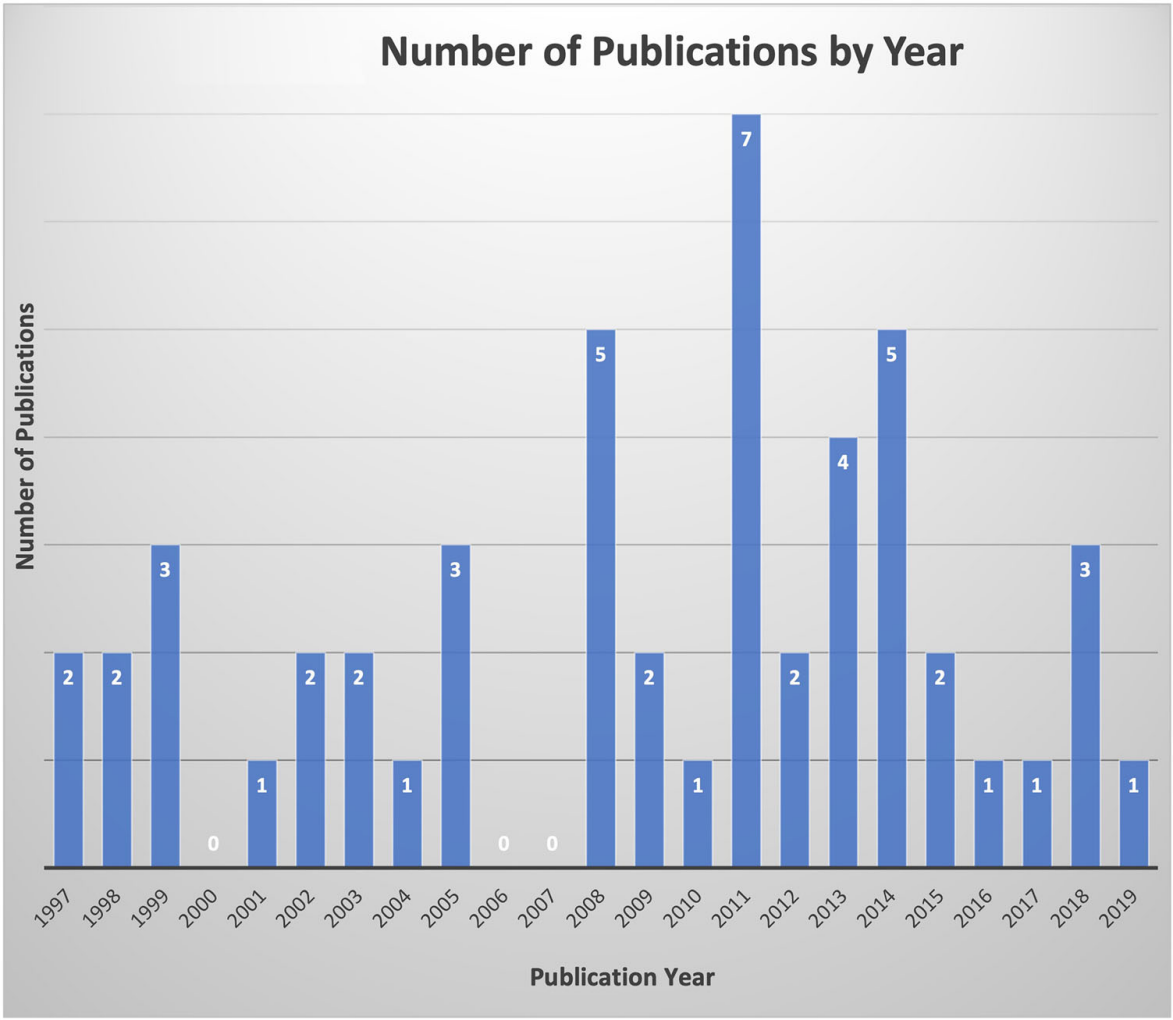
Number of publications by year

**Fig. 4:**
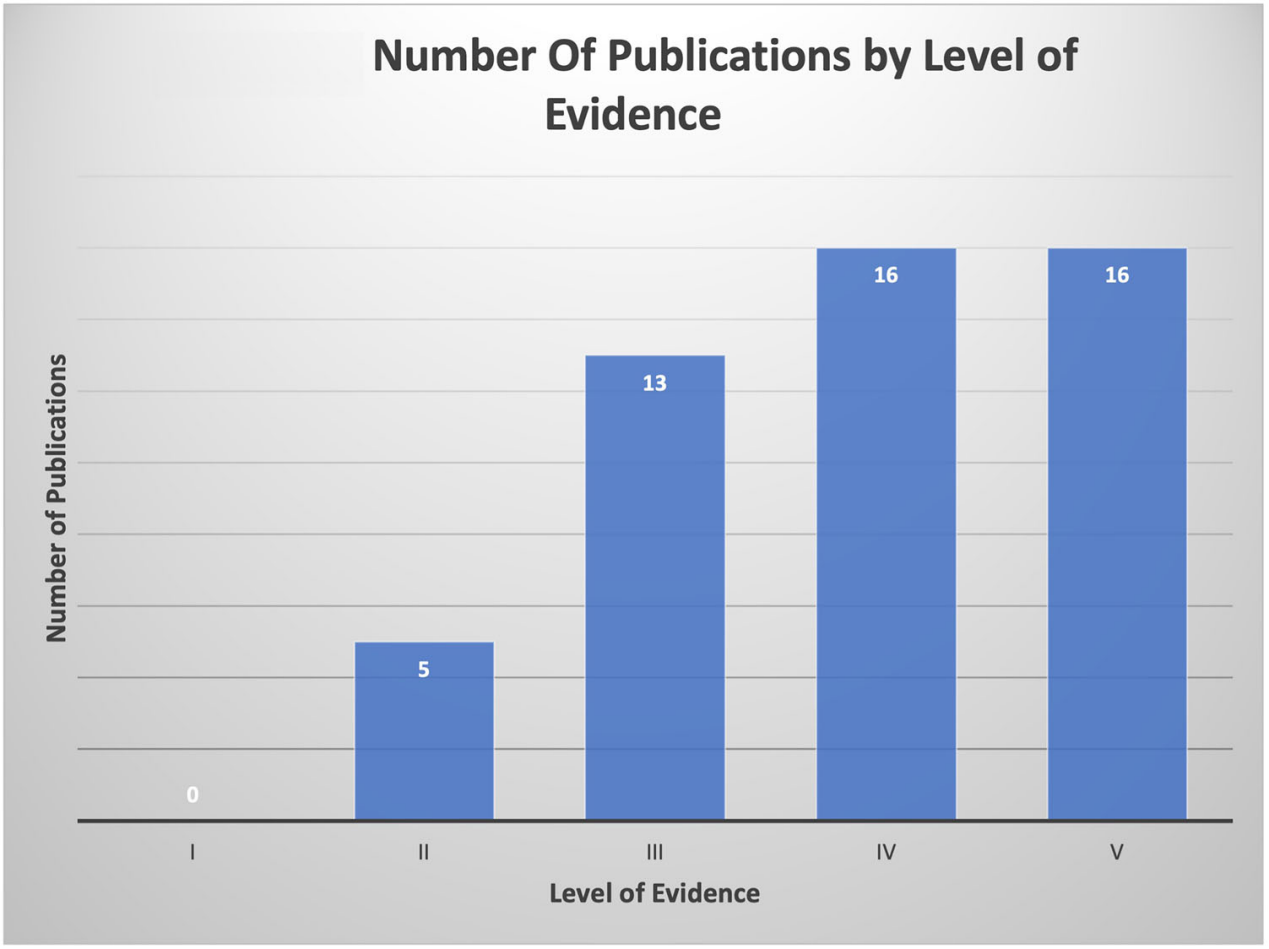
Number of publications by level of evidence

**Fig. 5:**
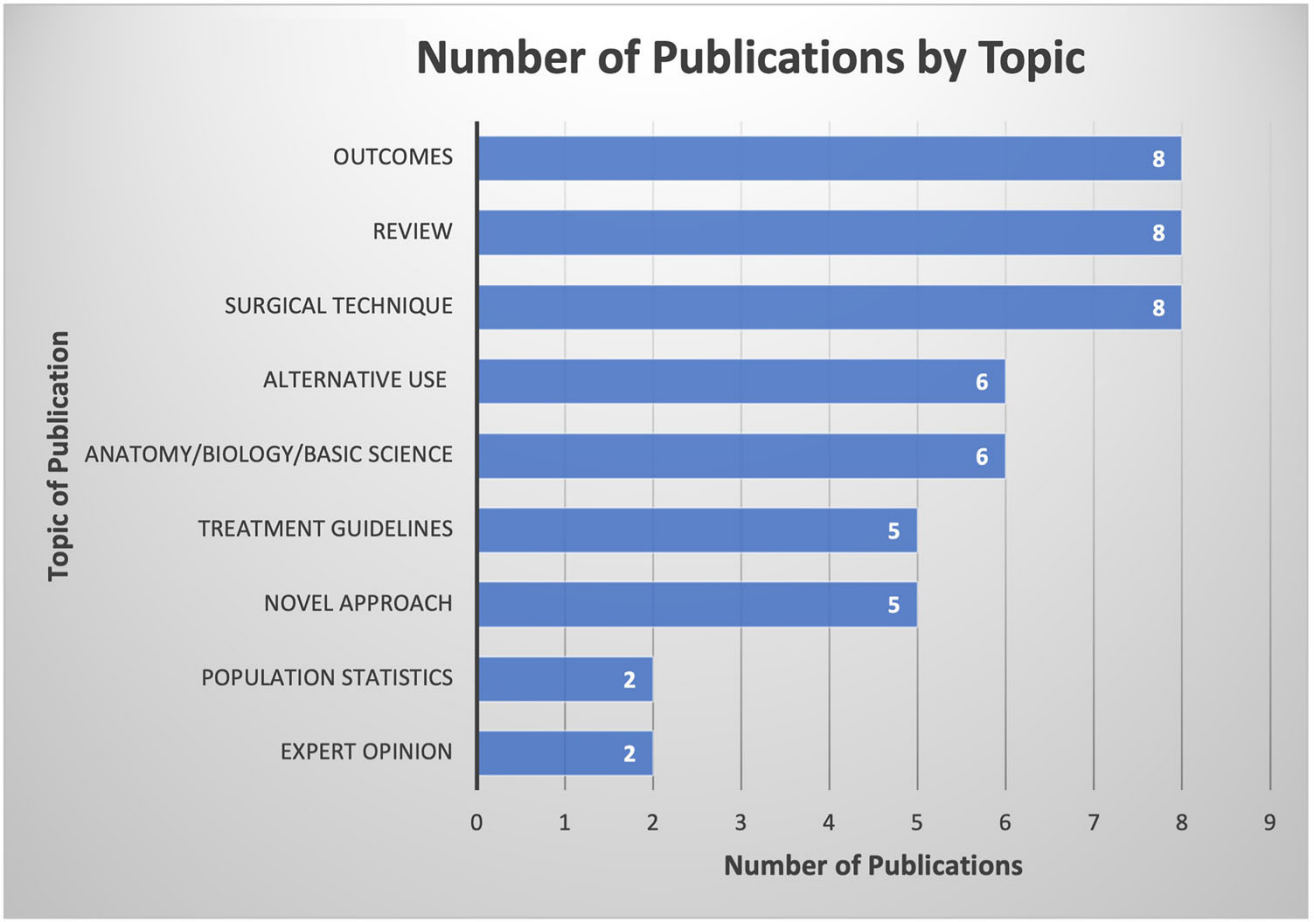
Number of publications by topic

**Fig. 6:**
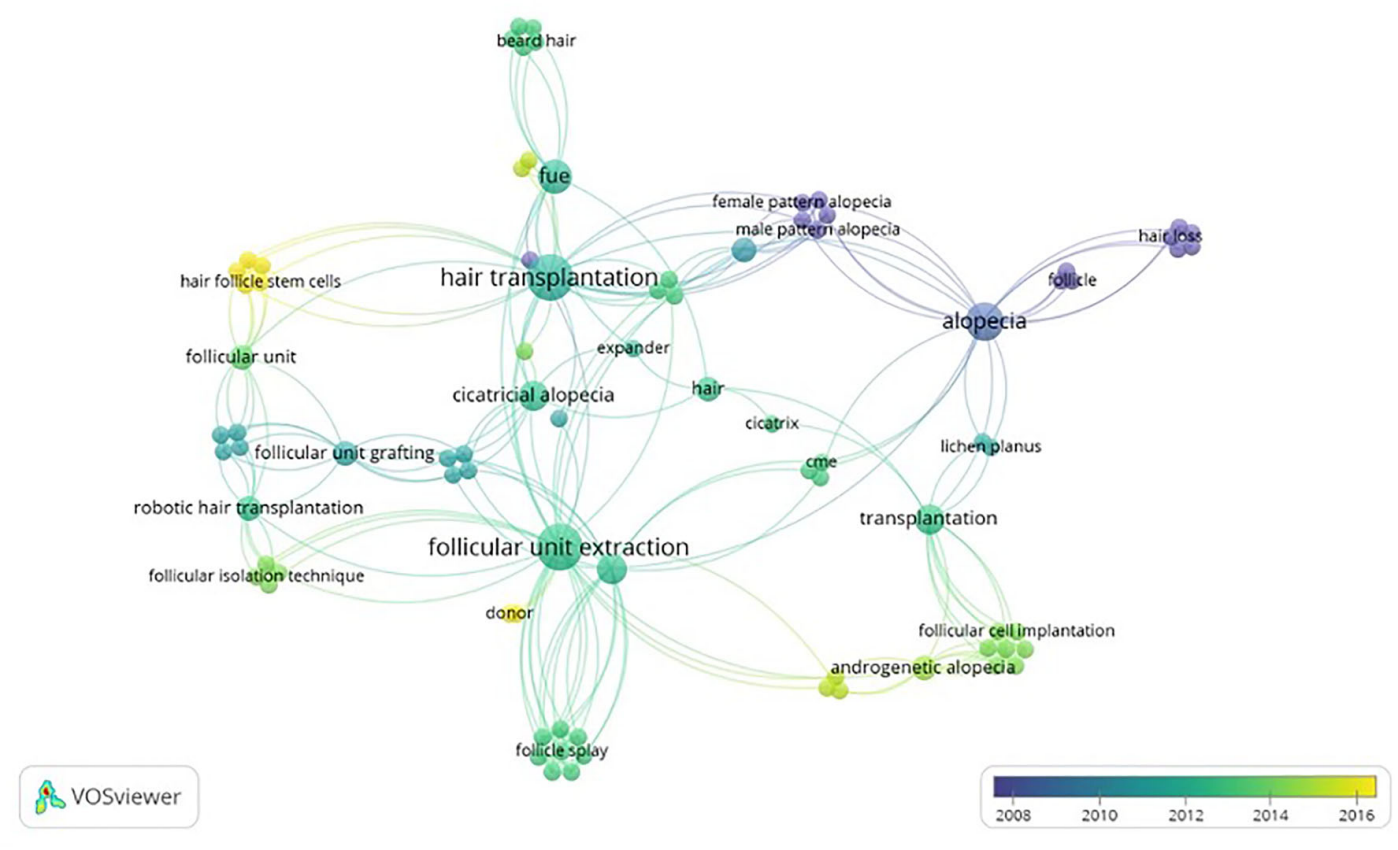
Co-occurrence map of keywords related to hair transplantation with overlay visualization

Publications included for the final analysis for the 50 most-cited articles pertaining to hair transplantation surgery were cited a total of 1341 times, with an average of 26.82 citations per article. Two authors had seven publications attributed to them, Bernstein and Rassman (Table 1), Rose was the second-most involved author with four publications. Figure 1 demonstrates the co-authorship association between the authors analyzed in this study. Cornell University was affiliated with the most publications (*n *= 6), with Columbia having the second most (*n *= 4) (Table 2). The USA was the country that produced the most publications (n = 25), and South Korea and Turkey produced the second most (*n *= 5) (Fig. 2). Most recently published study in the analysis was published in 2019, the oldest in 1997. In 2011, the year with the most attributable publications, seven studies were published. The second-most prolific years included 2008 and 2014. Each had five publications. Between 1997 and 2019, the years 2000, 2006, and 2007 had zero publications (Fig. 3). Of the 50 publications analyzed, no studies had a LOE of I; most studies were either LOE IV (*n *= 16) or V (*n *= 16) (Fig. 4). Of the chosen topics to stratify the publications, “Outcomes” (*n *= 8), “Review” (*n *= 8), and “Surgical Technique” (n = 8) were the most common (Fig. 5). Co-occurrence map of keywords related to hair transplantation with overlay visualization is demonstrated in Fig. 6**.**

**Table 1:** Author frequency

Author	Number of papers
Bernstein, R.M.	7
Rassman, W.R.	7
Rose, P.T.	4
Avram, M.R.	3
Barrera, A.	3
Harris, J.A.	3
Jimenez, F.	3

Only author with three or more contributions are included. Order of authorship is not considered

**Table 2:** Contributing institutions

Name of institution	Location of institution	Number of articles
Cornell University	Ithaca, New York	6
Columbia University	New York, New York	4
New Hair Institute	New York, New York	3
Baylor College of Medicine	Houston, Texas	3

Multiple institutions may be attributed to a single publication. Only institutions with three or more contributions are included in this table

Three most-cited publications in terms of total citation count were Rassman et al.’s “Follicular unit extraction: minimally invasive surgery for hair transplantation” (*n *= 153), Bernstein et al.’s “Follicular transplantation. Patient evaluation and surgical planning” (*n *= 73), and Berstein et al.’s “The aesthetics of follicular transplantation” (n = 60); these three publications were published between 1997–2002 (Table 3).

**Table 3:** Top 50 most-cited publications

Rank	Publication	Total citations
1	Rassman WR, Bernstein RM, McClellan R, Jones R, Worton E, Uyttendaele H. Follicular unit extraction: minimally invasive surgery for hair transplantation. *Dermatol Surg*. Aug 2002.	153
2	Bernstein RM, Rassman WR. Follicular transplantation. Patient evaluation and surgical planning. *Dermatol Surg*. Sep 1997.	73
3	Bernstein RM, Rassman WR. The aesthetics of follicular transplantation. *Dermatol Surg*. Sep 1997.	60
4	Asakawa K, Toyoshima KE, Ishibashi N, et al. Hair organ regeneration via the bioengineered hair follicular unit transplantation. *Sci Rep*. 2012.	51
5	Jimenez F, Ruifernandez JM. Distribution of human hair in follicular units. A mathematical model for estimating the donor size in follicular unit transplantation. Dermatol Surg. Apr 1999.	50
6	Martinez ML, Escario E, Poblet E, et al. Hair follicle-containing punch grafts accelerate chronic ulcer healing: A randomized controlled trial. J Am Acad Dermatol. Nov 2016.	45
7	Barrera A. The use of micrografts and minigrafts in the aesthetic reconstruction of the face and scalp. *Plast Reconstr Surg*. Sep 2003.	38
8	Leavitt M, Perez-Meza D, Rao NA, Barusco M, Kaufman KD, Ziering C. Effects of finasteride (1 mg) on hair transplant. Dermatol Surg. Oct 2005.	38
9	Harris JA. Follicular unit extraction. Facial Plast Surg. Nov 2008.	33
10	Bernstein RM, Rassman WR. Follicular transplantation. Patient evaluation and surgical planning. Dermatol Surg. Sep 1997.	32
11	Avram M, Rogers N. Contemporary hair transplantation. *Dermatol Surg*. Nov 2009.	31
12	Avram MR, Watkins SA. Robotic follicular unit extraction in hair transplantation. *Dermatol Surg*. Dec 2014.	30
13	Rousso DE, Kim SW. A review of medical and surgical treatment options for androgenetic alopecia. *JAMA Facial Plast Surg*. Nov-Dec 2014.	30
14	Cole JP. An analysis of follicular punches, mechanics, and dynamics in follicular unit extraction. *Facial Plast Surg Clin North Am*. Aug 2013.	29
15	Bernstein RM, Rassman WR, Seager D, et al. Standardizing the classification and description of follicular unit transplantation and mini-micrografting techniques. The American Society for Dermatologic Surgery, Inc. *Dermatol Surg*. Sep 1998.	28
16	Onda M, Igawa HH, Inoue K, Tanino R. Novel technique of follicular unit extraction hair transplantation with a powered punching device. *Dermatol Surg*. Dec 2008.	25
17	Rose PT. The latest innovations in hair transplantation. *Facial Plast Surg*. Aug 2011.	25
18	Tsai RY, Lee SH, Chan HL. The distribution of follicular units in the Chinese scalp: implications for reconstruction of natural-appearing hairlines in Orientals. *Dermatol Surg*. Jun 2002.	25
19	Bernstein RM, Rassman WR. Follicular unit transplantation: 2005. *Dermatol Clin*. Jul 2005.	24
20	Vogel JE, Jimenez F, Cole J, et al. Hair restoration surgery: the state of the art. *Aesthet Surg J*. Jan 2013.	24
21	Jung JH, Rah DK, Yun IS. Classification of the female hairline and refined hairline correction techniques for Asian women. *Dermatol Surg*. Apr 2011.	23
22	Rose PT, Nusbaum B. Robotic hair restoration. *Dermatol Clin*. Jan 2014.	23
23	Bunagan MJ, Banka N, Shapiro J. Hair transplantation update: procedural techniques, innovations, and applications. *Dermatol Clin*. Jan 2013.	22
24	Umar S. Hair transplantation in patients with inadequate head donor supply using nonhead hair: report of 3 cases. *Ann Plast Surg*. Oct 2011.	22
25	Avram MR, Finney R, Rogers N. Hair Transplantation Controversies. *Dermatol Surg*. Nov 2017.	21
26	Jung S, Oh SJ, Hoon Koh S. Hair follicle transplantation on scar tissue. *J Craniofac Surg*. Jul 2013.	21
27	Lee SJ, Lee HJ, Hwang SJ, et al. Evaluation of survival rate after follicular unit transplantation using the KNU implanter. *Dermatol Surg*. Aug 2001.	19
28	Crisostomo MR, Crisostomo MC, Crisostomo MG, Gondim VJ, Crisostomo MR, Benevides AN. Hair loss due to lichen planopilaris after hair transplantation: a report of two cases and a literature review. *An Bras Dermatol*. Mar-Apr 2011.	18
29	Harris JA. New methodology and instrumentation for follicular unit extraction: lower follicle transection rates and expanded patient candidacy. *Dermatol Surg*. Jan 2006.	18
30	Higgins CA, Christiano AM. Regenerative medicine and hair loss: how hair follicle culture has advanced our understanding of treatment options for androgenetic alopecia. *Regen Med*. Jan 2014.	18
31	Shao H, Hang H, Yunyun J, et al. Follicular unit transplantation for the treatment of secondary cicatricial alopecia. *Plast Surg (Oakv)*. Winter 2014.	18
32	Barr L, Barrera A. Use of hair grafting in scar camouflage. *Facial Plast Surg Clin North Am*. Aug 2011.	17
33	Patwardhan N, Mysore V, Force IDT. Hair transplantation: standard guidelines of care. *Indian J Dermatol Venereol Leprol*. Jan 2008.	17
34	Unger WP. Hair transplantation: current concepts and techniques. *J Investig Dermatol Symp Proc*. Dec 2005.	17
35	Liu YS, Jee SH, Chan JL. Hair transplantation for the treatment of lichen planopilaris and frontal fibrosing alopecia: A report of two cases. *Australas J Dermatol*. May 2018.	16
36	Umar S. Use of body hair and beard hair in hair restoration. *Facial Plast Surg Clin North Am*. Aug 2013.	16
37	Akdag O, Evin N, Karamese M, Tosun Z. Camouflaging Cleft Lip Scar Using Follicular Unit Extraction Hair Transplantation Combined with Autologous Fat Grafting. *Plast Reconstr Surg*. Jan 2018.	15
38	Gokrem S, Baser NT, Aslan G. Follicular unit extraction in hair transplantation: personal experience. *Ann Plast Surg*. Feb 2008.	15
39	Humayun Mohmand M, Ahmad M. Effect of Follicular Unit Extraction on the Donor Area. *World J Plast Surg*. May 2018.	15
40	Shin JW, Kwon SH, Kim SA, et al. Characteristics of robotically harvested hair follicles in Koreans. *J Am Acad Dermatol*. Jan 2015.	15
41	Bernstein RM, Rassman WR. The logic of follicular unit transplantation. *Dermatol Clin*. Apr 1999.	14
42	Kurata S, Ezaki T, Itami S, Terashi H, Takayusu S. Viability of isolated single hair follicles preserved at 4 degrees C. *Dermatol Surg*. Jan 1999.	14
43	Rousso DE, Presti PM. Follicular unit transplantation. *Facial Plast Surg*. Nov 2008.	14
44	Ergun SS, Sahinoglu K. Eyebrow transplantation. *Ann Plast Surg*. Dec 2003.	13
45	Gho CG, Martino Neumann HA. Donor hair follicle preservation by partial follicular unit extraction. A method to optimize hair transplantation. *J Dermatolog Treat*. Nov 2010.	13
46	Karacal N, Uraloglu M, Dindar T, Livaoglu M. Necrosis of the donor site after hair restoration with follicular unit extraction (FUE): a case report. *J Plast Reconstr Aesthet Surg*. Apr 2012.	13
47	Ors S, Ozkose M, Ors S. Follicular Unit Extraction Hair Transplantation with Micromotor: Eight Years Experience. *Aesthetic Plast Surg*. Aug 2015.	13
48	Woods R, Campbell AW. Chest hair micrografts display extended growth in scalp tissue: a case report. *Br J Plast Surg*. Dec 2004.	13
49	Gho CG, Neumann HA. Improved hair restoration method for burns. *Burns*. May 2011.	12
50	Sim JH, Park MJ, Park S, Lee ES. Altered expression of costimulatory molecules in Behcet's disease according to clinical activity. *Br J Dermatol*. Jun 2011.	12

When the publications were sorted by citation density (citation per year since publication year), Rassman et al.’s “Follicular unit extraction: minimally invasive surgery for hair transplantation” was the top publication again (*n *= 7.29). Martinez et al.’s study “Hair follicle-containing punch grafts accelerate chronic ulcer healing: A randomized controlled trial” had the second highest citation density (*n *= 6.43), and Asakawa et al.’s “Hair organ regeneration via the bioengineered hair follicular unit transplantation” had the third highest (*n *= 4.64). Nine of the 10 publications in the top 10 by citation density were published between 2012 and 2018 (Table 4).

**Table 4:** Top articles by citations per year since publication

Rank	Publication	Publication year	Citations per year since publication
1	Rassman WR, Bernstein RM, McClellan R, Jones R, Worton E, Uyttendaele H. Follicular unit extraction: minimally invasive surgery for hair transplantation. *Dermatol Surg*. Aug 2002.	2002	7.29
2	Martinez ML, Escario E, Poblet E, et al. Hair follicle-containing punch grafts accelerate chronic ulcer healing: A randomized controlled trial. *J Am Acad Dermatol*. Nov 2016.	2016	6.43
3	Asakawa K, Toyoshima KE, Ishibashi N, et al. Hair organ regeneration via the bioengineered hair follicular unit transplantation. *Sci Rep*. 2012.	2012	4.64
4	Avram MR, Finney R, Rogers N. Hair Transplantation Controversies. *Dermatol Surg*. Nov 2017.	2017	3.50
5	Avram MR, Watkins SA. Robotic follicular unit extraction in hair transplantation. *Dermatol Surg*. Dec 2014.	2014	3.33
6	Rousso DE, Kim SW. A review of medical and surgical treatment options for androgenetic alopecia. *JAMA Facial Plast Surg*. Nov-Dec 2014.	2014	3.33
7	Liu YS, Jee SH, Chan JL. Hair transplantation for the treatment of lichen planopilaris and frontal fibrosing alopecia: A report of two cases. *Australas J Dermatol*. May 2018.	2018	3.20
8	Akdag O, Evin N, Karamese M, Tosun Z. Camouflaging Cleft Lip Scar Using Follicular Unit Extraction Hair Transplantation Combined with Autologous Fat Grafting. *Plast Reconstr Surg*. Jan 2018.	2018	3.00
9	Humayun Mohmand M, Ahmad M. Effect of Follicular Unit Extraction on the Donor Area. *World J Plast Surg*. May 2018.	2018	3.00
10	Cole JP. An analysis of follicular punches, mechanics, and dynamics in follicular unit extraction. *Facial Plast Surg Clin North Am*. Aug 2013.	2013	2.90

## Discussion

Advancement in knowledge, technology, and surgical technique within the field of hair transplantation continues to promote better patient outcomes and minimize risks. Hair transplantation effectively improves the quality of life of patients suffering from various types of alopecia (e.g., androgenetic alopecia) and scarring caused by trauma or surgery. Indications continue to expand. By performing a bibliometric review on “hair transplant,” we identified the most-cited publications on the topic. Although this type of analysis makes it difficult to determine the quality of a publication, it quantitatively measures the level of influence that certain hair transplant publications have had on the scientific literature and, ultimately, clinical practice. This analysis aims to serve as a tool for surgeons, clinicians, and researchers to quickly identify the top-cited articles on hair transplantation rather than having to sift through the over 4000 published articles that exist today. Altogether, the top 50 publications on hair transplantation included for analysis have amassed over 1300 citations and serve as a snapshot for the most discussed ideas within the field. On average, the number of citations per publication included in our study, 26.82, is relatively low when compared to similar studies. For reference, bibliometric reviews analyzing facial rejuvenation, facial plastic surgery, and classic plastic surgery studies had average citation rates of 138, 150, and 859, respectively [8–10]. This may be attributed to the fact that these bibliometric reviews covered broader topics and/or publications that have been in circulation for a longer period of time and thus have amassed higher citation counts. This further highlights the relative novelty of hair transplantation surgery.

It is interesting to note the temporal evolution in topics of popular hair transplant manuscripts. Articles published in earlier years tended to consist of expert opinion, surgical technique, and treatment guidelines for conventional hair restoration. Content aimed to improve graft survival, promote ideal esthetic results, characterize optimal graft sizes, and provide general standardization to hair transplant procedures. Logically, this content established the foundation from which the field of research surrounding hair transplantation could emerge. Using number of citations as a measure of influence, the present study demonstrates that newer publications pertaining to hair transplant surgery have been more influential. In general, one might expect for older publications to have higher citation rates. After all, the longer a publication has been in circulation, the more likely it is to be cited. However, the opposite seems to be true when examining hair transplantation research, a result that may be explained by the content of newer studies.

Particularly after the year 2011, which happens to be the year with the highest number of influential publications, we saw an emergence of articles discussing alternative uses and novel approaches for hair transplantation. Researchers began to expand the utility of hair transplantation for new applications such as scar concealment, wound healing, and burns treatment [11–15]. Three influential articles on the implementation of robotic-assisted follicular unit extraction for hair transplantation were published between 2014–2015 [16–18]. Prospect of enhanced precision and efficiency with robotics proved enticing for both patients and providers. Overall, the predominant topic, as illustrated by the co-occurrence visualization of publication keywords, was follicular unit extraction. This keyword is intertwined with several topics of hair transplantation research including etiology of hair loss (i.e., cicatricial alopecia and androgenic alopecia) and technique (i.e., robotic hair transplantation and follicular unit grafting). Keyword co-occurrence analysis allows one to discover nodes of the literature interests and gauge possible future research topics. From the analysis presented in the present study, follicular unit extraction is a topic that may continue to garner attention in the field of hair transplantation.

Rassman, W.R. and Bernstein, R.M., two specialists in hair restoration surgery from New York that collaborated on several manuscripts, stand out as the authors with both the highest number of total publications (*n *= 7) and the top three most-cited articles on hair transplantation [4–6]. They also represented the largest nodes for co-citation analysis, demonstrating that their influence on the hair transplantation literature permeates through other prominent researchers in the field. They became recognized for pioneering their technique of Follicular Unit Extraction (FUE). They performed thousands of “micrografts” within a single session. This seminal work refined hair transplantation and brought it into the modern era by enabling surgeons to make smaller and less invasive incisions. As a result, more follicular unit grafts could be placed within a given area. They also were notable for their contributions to patient assessment and surgical planning with consideration of esthetic results. Their works set the stage for further innovation within the field of hair transplantation.

In analyzing the level of evidence of the top 50 most-cited articles on hair transplantation, only 10% (*n *= 5) of the studies were found to have Level II evidence. No articles managed to provide Level I evidence. Within the articles with Level II evidence, Leavitt et al. studied the effects of finasteride (1 g) on hair transplant. Martinez et al. substantiated the impact of hair follicle-containing punch grafts on chronic ulcer healing. Shao et al. employed hair transplantation for treatment of secondary cicatricial alopecia. Ors et al. used micromotor for follicular unit extraction hair transplantation. Bernstein and Rassman compared dissecting microscope against magnifying loupes with transillumination for follicular unit graft preparation [14, 19–22]. Median level of evidence among the top 50 articles was Level IV, mainly case-series or case-control studies. There was relatively even distribution of studies between Levels III–V. Overall, the level of evidence was skewed toward weaker studies. An apparent paucity of impactful, high level of evidence research concerning surgical technique in hair transplantation persists. In our opinion, the current deficit in systematic review and meta-analysis of randomized controlled trials likely stems from the challenges of conducting randomized controlled trials for reasons related to recruitment, cost, time investment, and ethics.

The US proves to be the predominant country regarding publication of high-impact articles related to hair transplantation. Compared to the runner-ups, Turkey and South Korea, which each claim five of the top 50 most-cited publications, the US has contributed 25 of the top 50 publications. This discrepancy between countries may be attributed to several causes. The US is home to some of the most prestigious international hair transplant medical associations and conferences, for example, the American Board of Hair Restoration Surgery (ABHRS), first organized in 1995. It continues as the only internationally recognized medical association granting board certification for hair restoration surgery [23]. Another possible explanation is that non-English manuscripts may not be adequately represented by citation indexes such as Web of Science. Although we did not restrict our search by language, it is possible that works from other countries are being overlooked. Finally, the US, between 1996–2020, contributed more total publications to scientific research than any other country [24]. It is unsurprising that this trend carries over to a specific topic such as hair transplantation.

New York State stands out as the world’s epicenter of prominent hair transplantation research. Institutions such as Cornell University (*n *= 6), Columbia University (*n *= 4), and New Hair Institute (*n *= 3) have contributed a combined 13 out of the 25 highly influential publications from the US. Baylor College of Medicine (*n *= 3) in Texas stands out as another prolific institution. No other institutions contributed three or more publications to the most-cited literature.

## Limitations

Although bibliometric analysis provides valuable insight into the research landscape of a particular topic, it holds several limitations. Using a citation database such as Web of Science may lead to bias in data source, for not all relevant publications are necessarily captured. Authors carefully crafted their search criteria to find all highly cited manuscripts; however, omission of influential work remains a real possibility. Works published in non-indexed journals, lectures, textbooks, or digital media may provide high-quality information but are not considered by our search. Publications in languages other than English were also excluded. This may have affected publication density by country. Citation counts do not always convey the level of importance of a scientific publication or its ability to impact future inquiry and practice. Citation counts are subject to artificial inflation through certain practices such as self-citation, citation stacking aka “citation cartels,” and sub-publication. Recently published articles have not had enough time to garner a large number of total citations. Therefore, they may have been excluded from our top 50 analysis despite their possible distinction.

## Conclusion

This bibliometric review provides an important roadmap to the most influential publications regarding hair transplantation. Our analysis highlights the transformative work of authors Rassman and Bernstein, the emergence of follicular unit extraction as a groundbreaking procedure. More importantly, it identifies a shift in focus toward alternative uses and novel approaches in hair transplantation post-2011. The US emerged as a significant contributor to high-impact hair transplantation research, with notable institutions such as Cornell University, Columbia University, and New Hair Institute leading the quest. However, the analysis also revealed a critical need for contemporary high-level evidence-based research. It is apparent that many influential studies were found to have lower levels of evidence. While this review offers valuable insights into the field's evolution, it also underscores the ongoing need for the pursuit of high-quality, innovative research to further advance the understanding and practice of hair transplantation and continue to advance patient satisfaction and our clinical outcomes.
